# Automatic detection of beating cilia with frequencies estimations

**DOI:** 10.1186/2046-2530-4-S1-P85

**Published:** 2015-07-13

**Authors:** E Puybareau, H Talbot, G Pelle, B Louis, L Najman, A Coste

**Affiliations:** 1 Laboratoire d'Informatique Gaspard Monge, CNRS UMR8049, Université Paris-Est ESIEE, Marne-La-Vallée, France; 2 Inserm U955, Eq13, Créteil 94000, Créteil, France; 3 Université Paris-Est, Faculté de Médecine, Créteil 94000, Créteil, France; 4 CNRS ERL 7240, Créteil 94000, Créteil, France

## Objectives

Muco-ciliary clearance is the airway first mechanism of defence against environmental attacks such as micro-organisms or pollution. Cilia motility impairment can be either of genetic (primary ciliary dyskinesia) or acquired origin (environmental attacks), entailing chronic diseases. It is of interest for practitioners to evaluate cilia beating frequency easily, robustly and reliably. As yet, no fully automatized method is available.

## Methods

Ciliated cells were sampled in patients by brushing nasal mucosa and cilia beating was recorded using high speed video microscopy. We first estimated and removed the sensor pattern. We then stabilized the sequence assuming rigid transforms. We retained only the moving parts of the sequence and, after deblurring, characterized and segmented the moving parts in several regions of interest. The frequency was estimated for each region.

## Results

We output the processed sequence, a labeled mask of the various beating zones and a chart of the frequency observed in each region. Hence we obtained synchronization information between the different parts of the observed ciliated cells. An estimation of frequencies for each beating part is the final result.

## Conclusion

With this new method, we propose a fully automatic estimation of cilia beating frequencies, which is able to deal with acquisition artifacts, such as sensor patterns, vibrations and noise, but also with the variety of frequencies we can observe on a single sample. We believe this may be a useful method for practitioners.

